# Interleukin gene polymorphisms and alopecia areata: A systematic review and meta-analysis

**DOI:** 10.1097/MD.0000000000037300

**Published:** 2024-02-23

**Authors:** Zasia Hossain Tishe, Sanjana Shawkat, Meherun Nessa Popy, Ashfaq Ahmed, Sadia Biswas Mumu, Mohd Nazmul Hasan Apu, Md Shaki Mostaid

**Affiliations:** a Department of Pharmaceutical Sciences, Faculty of Health and Life Sciences, North South University, Dhaka, Bangladesh; b Department of Clinical Pharmacy and Pharmacology, Faculty of Pharmacy, University of Dhaka, Dhaka, Bangladesh.

**Keywords:** alopecia areata, IL2RA, interleukin, meta-analysis, rs31184701

## Abstract

**Background:**

Alopecia areata (AA) is an autoimmune disease which results in non-scarring hair loss on the scalp or any surface with hair. Several genetic polymorphisms of the interleukin genes have been linked with this disease but the results are inconsistent. This systematic review and meta-analysis were done to find the association between rs3118470, rs2275913, rs3212227, and rs10889677 of the IL2RA, IL17A, IL12B, and IL23R genes, respectively, of the interleukin family with alopecia areata.

**Methods:**

A comprehensive search for relevant research articles was conducted in Pubmed, Google Scholar, and Embase databases. Our search yielded 8 relevant articles with 1940 cases and 1788 controls. The odds ratio with 95% confidence intervals was calculated using fixed effect and random effect models. Heterogeneity was determined using the Q-test and *I*^2^ test. Publication bias was determined and funnel plots were used to adjust the odds ratio.

**Results:**

We found a significant risk effect for rs3118470 of the IL2RA gene with alopecia areata in the dominant model (CC + CT vs TT; OR = 1.54, 95% confidence interval = 1.05–2.26, *P* < .05, *I*^2^ = 69.03%) and homozygous model (CC vs TT; OR = 2.00, 95% confidence interval = 1.07–3.71, *P* < .05, *I*^2^ = 72.84%). For the other single nucleotide polymorphisms, we could not find any statistically significant association with the disease.

**Conclusion:**

Our analysis showed that mutation of rs3118470 of IL2RA gene possesses a significant risk effect for alopecia areata. Future studies with larger sample sizes and ethnic backgrounds are warranted to confirm our findings.

## 1. Introduction

Alopecia areata (AA) is an autoimmune disease that is characterized by non-scarring hair loss on the scalp or any surface with hair. The clinical signs of AA differ from little well-defined patches of balding to the diffuse inclusion of the scalp or the whole body.^[1]^ Each patient experiences a unique and unpredictable course, and it is categorized into the following 3 groups based on its severity and the areas of hair loss: (1) alopecia areata in patches, the most common type, is characterized by round or oval patches on the head or other parts of the body in 90% of cases; (2) alopecia totalis, in which the scalp is completely or nearly completely devoid of hair; and (3) alopecia universalis, the most severe condition that stands out from the other 2 due to the fact that hair loss affects the entire body in addition to the scalp and face.^[2]^ At present, approximately 2% of the general population, across all racial, gender, and age groups, has been affected by AA.^[1]^ It can affect people of all ages, however, the prevalence of occurring alopecia areata seems higher in children (1.92%) than in adults (1.47%).^[3]^ The immune response, genetic and environmental factors play a very important role in the onset and progression of alopecia areata.^[4,5]^

AA is linked with atopic diseases, and this condition’s co-existence with atopic eczema and loss of function mutation of the Filaggrin gene may result in a more severe form of AA.^[6–8]^ Genetic polymorphism plays a crucial role in the onset of AA.^[9]^ Studies have identified specific genetic polymorphisms of CTLA4, HLA, IL2RA, and IL13 genes associated with the disease, and AA patients frequently have family histories of autoimmune-related disorders.^[10,11]^ Several studies have found a significant association of interleukin genes with AA.^[12–14]^ Overexpression of interleukin gene IL-1β is found in the human scalp areas affected by AA, especially in the early stages of the condition.^[15]^

In this present study, we focused on interleukins (ILs) which play an important role in the pathogenesis of AA. Among these interleukins, IL2RA, IL17A, IL12B, and IL23R have been reported as the most prominent genes linked to alopecia areata by several case–control studies.^[16–20]^ Polymorphisms of IL2RA may affect the pathogenesis of alopecia areata as it is highly expressed on CD4 + CD25 + regulatory T cells and plays a vital role in immune homeostasis and suppression of autoimmune response.^[19]^ An intronic SNP rs3118470 located on the IL2RA, was reported to be significantly associated with AA in the Chinese and Iranian population.^[19,27]^ However, it showed no significant association in the population from Jordan and Caucasian ancestry.^[16,21]^

The IL17A rs2275913 polymorphism, located in the promoter region of IL17A gene was found to be significantly associated with AA in the population of Egypt.^[28]^ However, there was no significant association found in the Iranian and Jordanian populations.^[17,20]^

Moreover, rs3212227, located in the 3´ untranslated region of IL12B gene was reported to have a significant association with the risk of developing AA in the Iranian population.^[17]^ However, there was no significant association found with AA in the population from Turkey and Jordan.^[18,20]^

Lastly, rs10889677, located in the 3′ untranslated region of the IL23R was found to be significantly associated with AA in the population of Iran.^[17]^ However, there was no significant association shown in the Turkish and Jordanian populations.^[16,18]^

To reconcile the conflicting findings in the case–control studies, we performed a meta-analysis on 4 SNPs rs3118470, rs2275913, rs3212227, and rs10889677 of the IL2RA, IL17A, IL12B, and IL23R genes, respectively, for a potential association with AA. We excluded other polymorphisms of interleukin genes due to the insufficient number of case-control studies for a retrospective analysis.

## 2. Methodology

### 2.1. Identification of relevant studies

A comprehensive search was conducted on PubMed, Google Scholar, and Embase databases to find relevant articles. The duration of the literature search was 4 months. The queries used for finding the articles in different databases are given in Table 1. Our systematic review and meta-analysis were performed adhering to the PRISMA-P guidelines.^[22]^

**Table 1:** Search query and articles that were found.

PubMed	(IL2RA molecule OR IL2RA) AND (polymorphism OR mutation OR variant) AND “Alopecia Areata’’ (IL17A molecule OR IL17A) AND (polymorphism OR mutation OR variant) AND “Alopecia Areata’’ (IL12B molecule OR IL12B) AND (polymorphism OR mutation OR variant) AND “Alopecia Areata’’ (IL23R molecule OR IL23R) AND (polymorphism OR mutation OR variant) AND “Alopecia Areata’’	19
Google scholar	IL2RA polymorphism mutation variant mutation OR variant OR polymorphism “ Alopecia Areata’’ IL17A polymorphism mutation variant mutation OR variant OR polymorphism “ Alopecia Areata’’ IL12B polymorphism mutation variant mutation OR variant OR polymorphism “ Alopecia Areata’’ IL23R polymorphism mutation variant mutation OR variant OR polymorphism “ Alopecia Areata’’	966
Embase	‘IL2RA AND polymorphism AND mutation AND variant AND “Alopecia Areata’’ AND (mutation OR variant OR polymorphism)’ ‘IL17A AND polymorphism AND mutation AND variant AND “Alopecia Areata’’ AND (mutation OR variant OR polymorphism)’ ‘IL12B AND polymorphism AND mutation AND variant AND “Alopecia Areata’’ AND (mutation OR variant OR polymorphism)’ ‘IL23R AND polymorphism AND mutation AND variant AND “Alopecia Areata’’ AND (mutation OR variant OR polymorphism)’	14

### 2.2. Inclusion criteria and exclusion criteria

The inclusion criteria of the research articles to be included in our meta-analysis were: (1) the association between IL2RA (rs3118470), IL17A (rs2275913), IL12B (rs3212227), and IL23R (rs10889677) gene polymorphisms with the risk of alopecia areata, (2) it must be a case-control study with genotypic frequencies. The exclusion criteria were: (1) review papers and animal model studies, (2) studies with incomplete genotype data, and (3) studies with other genes and polymorphisms related to alopecia areata.

### 2.3. Data extraction procedure

For relevant articles, 3 authors independently searched from the databases, and any discrepancies were discussed. The data included the author’s name followed by the year, number of cases and controls, and genotype data of case and control groups of the individual studies (Table 2). The quality of selected articles was evaluated using “The Newcastle-Ottawa scale” (Table 3).

**Table 2:** Characterization of included studies for the meta-analysis.

rs3212227 (IL12B)																							
Author	Year	Population				Allele (case)		Allele (control)			Genotype (case)				Genotype (control)								
		Case	Control			A allele	C allele	A allele		C allele	A/A	A/C	C/C		A/A	A/C		C/C					
Aytekin	2015	100	71			165	35	107		35	69	27	4		38	31		2					
Tabatabaei-Panah	2020	60	60			73	47	105		15	22	29	9		50	5		5					
AL-Eitan	2022	152	150			204	100	205		91	70	64	18		71	63		14					
**rs10889677 (IL23R**)																							
**Author**	**Year**	**Population**			**Allele (case**)				**Allele (control**)				**Genotype (case**)					**Genotype (control**)					
		**Case**		**Control**	**C** **allele**			**A allele**	**C allele**		**A allele**		**C/C**	**C/A**			**A/A**	**C/C**		**C/A**	**A/A**		
Aytekin	2015	100		71	90			110	75		67		20	50			30	22		31	18		
Tabatabaei-Panah	2020	60		60	61			59	84		36		21	19			20	27		30	3		
Alghamdi	2022	152		150	185			117	177		115		59	67			25	55		67	24		
**rs3118470 (IL2RA**)																							
**Author**	**Year**	**Population**						**Allele (case**)			**Allele (control**)			**Genotype (case**)							**Genotype (control**)		
		**Case**			**Control**			**T allele**	**C allele**		**T allele**		**C allele**	**T/T**				**T/C**	**C/C**		**T/T**	**T/C**	**C/C**
Redler	2012	768			658			952	584		895		423	295				362	111		304	287	68
Miao	2013	427			430			510	344		592		268	152				206	69		189	214	27
Moravvej	2018	69			69			94	44		123		15	39				16	14		59	5	5
Alghamdi	2022	152			150			77	227		60		236	9				59	84		6	48	94
**rs2275913 (IL17A**)																							
**Author**	**Year**	**Population**						**Allele (case**)			**Allele (control**)			**Genotype (case**)							**Genotype (control**)		
		**Case**			**Control**			**G allele**	**A allele**		**G allele**		**A allele**	**G/G**				**G/A**	**A/A**		**G/G**	**G/A**	**A/A**
Tabatabaei-Panah	2020	60			60			108	12		102		13	52				4	4		51	5	4
Seleit	2021	60			40			42	78		54		26	12				18	30		24	6	10
AL-Eitan	2022	152			150			215	89		219		75	78				59	15		85	49	13

**Table 3:** Quality assessment using Newcastle–Ottawa scale.

Author	Year	Selection				Comparability	Exposure			Total
		1	2	3	4	5	6	7	8	
Redler	2012	*	*	*	*	*		*	*	*******
Miao	2013	*	*	*	*	*		*	*	*******
Aytekin	2015	*	*	*	*	*		*	*	*******
Moravvej	2018	*	*	*	*	*		*	*	*******
Tabatabaei-Panah	2020	*	*	*	*	*		*	*	*******
Seleit	2021	*	*	*	*	*		*		******
Alghamdi	2022	*	*	*	*	*		*		******
AL-Eitan	2022	*	*	*	*	*		*		******
1 = Adequate case definition										
2 = Representativeness of the cases										
3 = Selection of controls										
4 = Definition of controls										
5 = Comparability of cases and controls on the basis of the design or analysis										
6 = Ascertainment of exposure										
7 = Same method of ascertainment for cases and controls										
8 = nonresponse rate										

### 2.4. Statistical analysis

Meta-analysis was performed using the Comprehensive Meta-Analysis version 3 Software (Englewood, NJ). The odds ratio (OR) with a 95% confidence interval (CI)^[23]^ was assessed to estimate the strength of association between IL2RA (rs3118470), IL17A (rs2275913), IL12B (rs321222)7, and IL23R (rs10889677) gene polymorphisms and risk of AA. To evaluate the heterogeneity, we performed Cochran Q-test, and its magnitude was determined by *I*^2^ test. When there was low (*I*^2^ < 25%) to moderate heterogeneity (*I*^2^ < 50%) we used the fixed effect model, otherwise random effects model^[24]^ was used for the calculation of odds ratio with 95% CI.

For rs3118470 (IL2RA), Allelic Model (C vs T), Dominant Model (CC + CT vs TT), Recessive Model (CC vs CT + TT), Heterozygous Model (CT vs TT), and Homozygous Model (CC vs TT) were assessed. For rs2275913 (IL17A), Allelic Model (A vs G), Dominant Model (AA + AG vs GG), Recessive Model (AA vs AG + GG), Heterozygous Model (AG vs GG), and Homozygous Model (AA vs GG) were assessed. For rs3212227 (IL12B), Allelic Model (C vs A), Dominant Model (CC + CA vs AA), Recessive Model (CC vs CA + AA), Heterozygous Model (CA vs AA), and Homozygous Model (CC vs AA) were assessed. For rs10889677 (IL23R), Allelic Model (A vs C), Dominant Model (AA + AC vs CC), Recessive Model (AA vs AC + CC), Heterozygous Model (AC vs CC), and Homozygous Model (AA vs CC) were assessed.

Publication bias was examined by Begg-Mazumdar rank correlation test and Egger regression test.^[25,26]^ For publication bias, we used the funnel plots to adjust the odds ratio. Two-tailed *P*-values were reported and all statistical tests were considered significant at *P* < .05.

## 3. Result

### 3.1. Characteristics of the published studies

**Figure 1.**
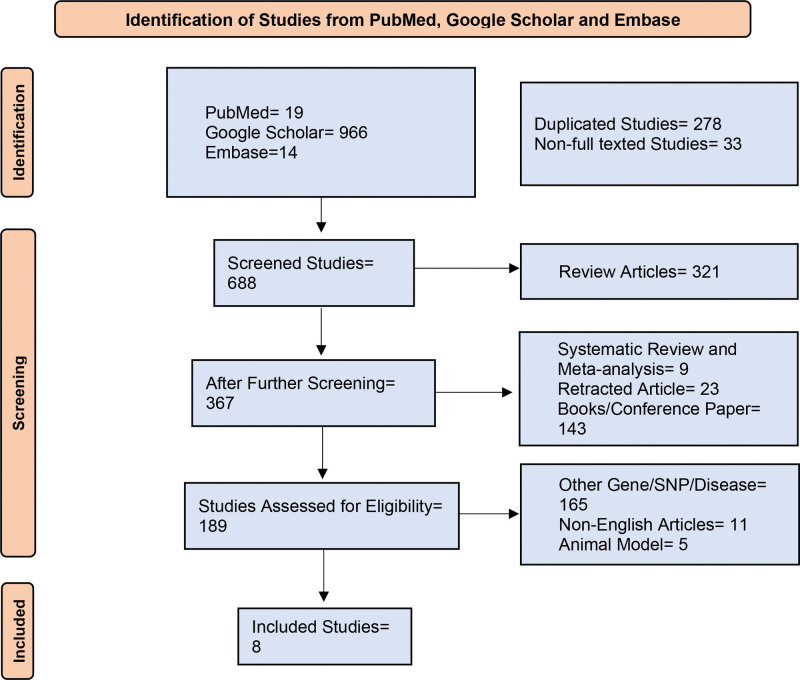
Flowchart showing the selection of included studies. The initial search results were 999 articles (PubMed = 19; Google Scholar = 966; Embase = 14). After a thorough screening, data from 8 articles were collected.

After a thorough screening, 8 articles were included in our meta-analysis. The selection procedure of included studies in our meta-analysis is shown by a flow diagram (Fig. 1). The initial search yielded a total of 999 articles from PubMed (n = 19), Google Scholar (n = 966), and Embase (n = 14). After the initial search, 278 duplicate studies and 33 non-full texted studies were excluded. In the screening procedure, 321 nonrelevant and review articles, 9 meta-analyses, 23 retracted, and conference papers were omitted. The assessment based on eligibility was done on 189 articles. Throughout the assessment, 165 articles were not related to Alopecia Areata and IL2RA, IL17A, IL12B IL23R genes, 11 were non-English publications and 5 articles were on animal models.

### 3.2. Meta-analysis of IL2RA rs3118470 polymorphism on AA risk

**Figure 2.**
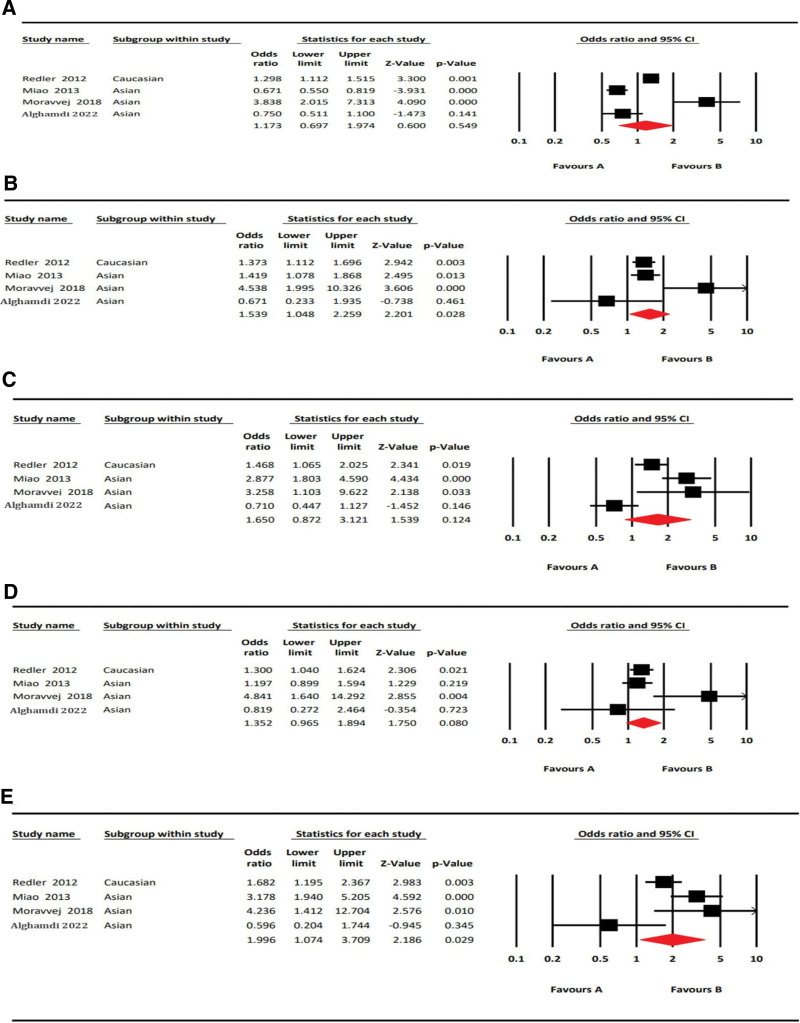
Forest plots of rs3118470 (IL2RA) with AA risk in 5 genetic models. A: Allelic Model (A vs G); B: Dominant Model (AA + AG vs GG); C: Recessive Model (AA vs AG + GG); D: Heterozygous Model (AG vs GG); E: Homozygous Model (AA vs GG).

All of the results obtained from the meta-analysis on the association between AA and IL2RA rs3118470 SNP are listed in Table 4. In total, 4 articles, with 1416 patients in the case and 1307 in the control group, were considered for the meta-analysis.^[16,19,21,27]^ Among these 4 articles, one was conducted on the Caucasian population, while 3 studies were on the Asian population. We found that IL2RA rs3118470 polymorphism confers a risk effect in the following models: dominant (CC + CT vs TT; OR = 1.54, 95%CI = 1.05–2.26, *P* < .05, *I*^2^ = 69.03%); homozygous (CC vs TT; OR = 2.00, 95%CI = 1.07–3.71, *P* < .05, *I*^2^ = 72.84%). However, allelic (C vs T; OR = 1.17, 95%CI = 0.70–1.97, *P* = .55, *I*^2^ = 93.34%); recessive (CC vs CT + TT; OR = 1.65, 95%CI = 0.87–3.12, *P* = .12, *I*^2^ = 84.61%); heterozygous (CT vs TT; OR = 1.35, 95%CI = 0.97–0.1.89, *P* = .08, *I*^2^ = 55.90%] models did not show statistically significant association. The forest plots are shown in Figure 2. According to the ethnicity mentioned in the articles, the populations from China, Iran, and Jordan were grouped as Asians, and the study with the German population was of Caucasian ethnicity.

**Table 4:** Association of rs3118470 (IL2RA) with alopecia areata.

Model name	Association test			Heterogeneity test			Publication bias (*P* value)	
	Odds ratio (OR)	95% CI	*P* value	Model	*I* ^2^	*P* value	Egger test	Begg-Mazumdar test
rs3118470 (IL2RA)								
Allelic model (C vs T)	1.17	0.70–1.97	.55	Random	93.34	.00	0.81	1.00
Dominant model (CC + CT vs TT)	1.54	1.05–2.26	**.03**	Random	69.03	.02	0.72	1.00
Recessive model (CC vs CT + TT)	1.65	0.87–3.12	.12	Random	84.61	.00	0.71	0.50
Heterozygous model (CT vs TT)	1.35	0.97–1.89	.08	Random	55.90	.08	0.59	0.50
Homozygous model (CC vs TT)	2.00	1.07–3.71	**.03**	Random	72.84	.01	0.99	1.00

The bold values indicate *P* < 0.05.

### 3.3. Meta-analysis of IL17A rs2275913 polymorphism on AA risk

**Figure 3.**
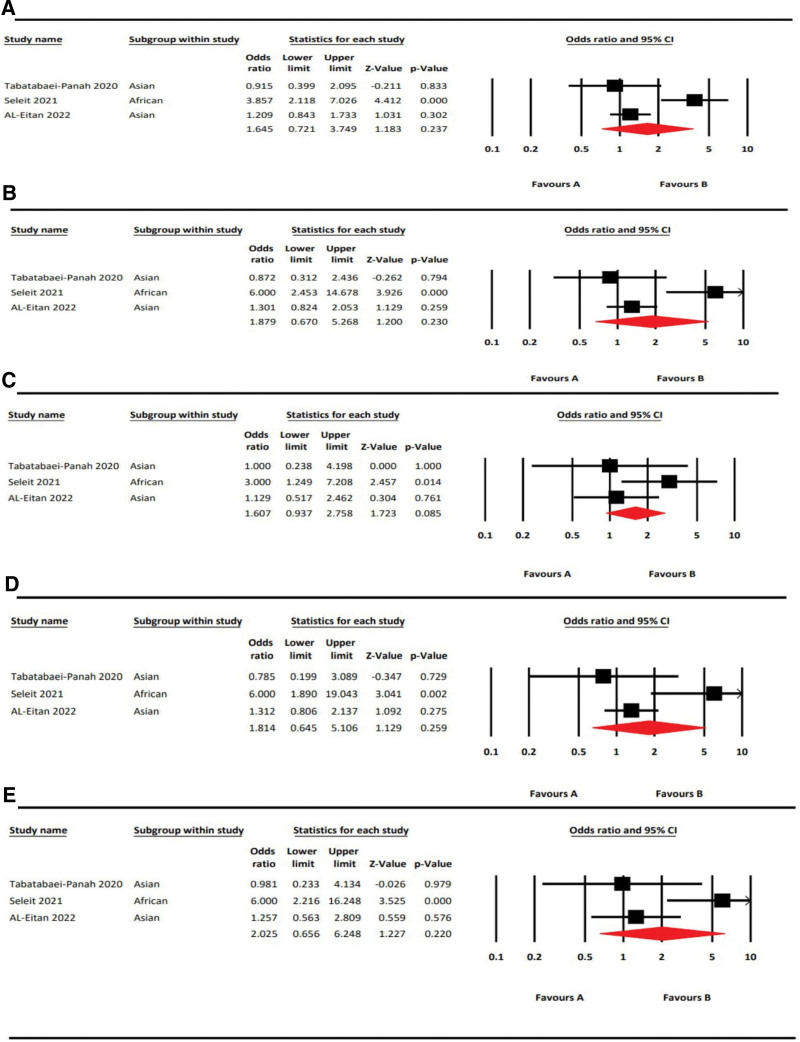
Forest plots of rs2275913 (IL17A) with AA risk in 5 genetic models. A: Allelic Model (A vs G); B: Dominant Model (AA + AG vs GG); C: Recessive Model (AA vs AG + GG); D: Heterozygous Model (AG vs GG); E: Homozygous Model (AA vs GG).

Table 5 summarizes the results of our meta-analysis of the association between the IL17A rs2275913 SNP and AA.^[17,20,28]^ Among 3 studies, 2 were conducted on the Asian population^[17,20]^; while the other was on the African (Egyptian) population.^[28]^ Our analysis showed IL17A rs2275913 polymorphism did not show a significant association in the allelic model, A vs G [OR = 1.65, 95%CI = 0.72–3.75, *P* = .24, *I*^2^ = 83.68%]; dominant model, AA + AG vs GG [OR = 1.88, 95%CI = 0.67–5.27, *P* = .23, *I*^2^ = 80.98%]; recessive model AA vs AG + GG [OR = 1.61, 95%CI = 0.94–2.76, *P* = .09, *I*^2^ = 36.65%], heterozygous model, AG vs GG [OR = 1.81, 95%CI = 0.65–5.11, *P* = .26, *I*^2^ = 70.02%]; homozygous model, AA vs GG [OR = 2.03, 95%CI = 0.66–6.25, *P* = .22, *I*^2^ = 70.98%]. The forest plots are shown in Figure 3.

**Table 5:** Association of rs2275913 (IL17A) with alopecia areata.

Model name	Association test			Heterogeneity test			Publication bias (*P* value)	
	Odds ratio (OR)	95% CI	*P* value	Model	*I* ^2^	*P* value	Egger test	Begg-Mazumdar test
rs2275913 (IL17A)								
Allelic model (A vs G)	1.65	0.72–3.75	.24	Random	83.68	.00	0.83	0.60
Dominant model (AA + AG vs GG)	1.88	0.67–5.27	.23	Random	80.98	.01	0.76	0.60
Recessive model (AA vs AG + GG)	1.61	0.94–2.76	.09	Fixed	36.65	.21	0.87	0.60
Heterozygous model (AG vs GG)	1.81	0.65–5.11	.26	Random	70.02	.04	0.76	0.60
Homozygous model (AA vs GG)	2.03	0.66–6.25	.22	Random	70.98	.03	0.98	0.60

### 3.4. Meta-analysis of IL12B rs3212227 polymorphism on AA risk

**Figure 4.**
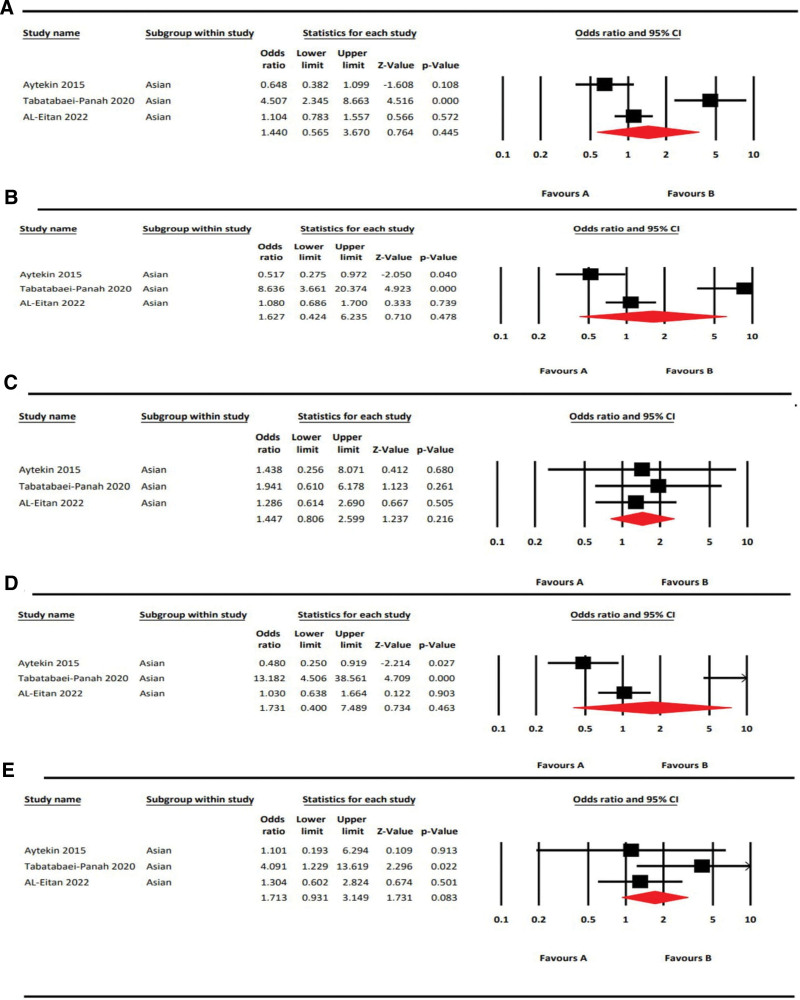
Forest plots rs3212227 (IL12B) of with AA risk in 5 genetic models. A: Allelic model (C vs A); B: Dominant model (CC + CA vs AA); C: Recessive model (CC vs CA + AA); D: Heterozygous model (CA vs AA) and E: Homozygous model (CC vs AA).

Table 6 summarizes the results of our meta-analysis of the association between the IL12B rs3212227 SNP and AA. Three articles were included in the meta-analysis consisting of 312 cases and 281 controls.^[17,18,20]^ All 3 of these studies were done on a population of Asian ethnicity. We found that IL12B rs3212227 polymorphism did not have any risk effect in the allelic model, C vs A [OR = 1.44, 95%CI = 0.57–3.67, *P* = .45, *I*^2^ = 90.56%]; dominant model, CC + CA vs AA [OR = 1.63, 95%CI = 0.42–6.24, *P* = .48, *I*^2^ = 92.69%]; recessive model CC vs CA + AA [OR = 1.45, 95%CI = 0.81–2.60, *P* = .22, *I*^2^ = 0%], heterozygous model, CA vs AA [OR = 1.73, 95%CI = 0.40–7.49, *P* = .46, *I*^2^ = 92.56%]; and homozygous model, CC vs AA [OR = 1.71, 95%CI = 0.93–3.15, *P* = .08, *I*^2^ = 29.95%]. The forest plots are shown in Figure 4. According to the ethnicity mentioned in the articles, the populations from Turkey, Iran, and Jordan were grouped as Asians.

**Table 6:** Association of rs3212227 (IL12B) with alopecia areata.

Model name	Association test			Heterogeneity test			Publication bias (*P* value)	
	Odds ratio (OR)	95% CI	*P* value	Model	*I* ^2^	*P* value	Egger test	Begg-Mazumdar test
rs3212227 (IL12B)								
Allelic model (C vs A)	1.44	0.57–3.67	.45	Random	90.56	.00	0.67	0.60
Dominant model (CC + CA vs AA)	1.63	0.42–6.24	.48	Random	92.69	.00	0.58	0.60
Recessive model (CC vs CA + AA)	1.45	0.81–2.60	.22	Fixed	0.00	.84	0.64	0.60
Heterozygous model (CA vs AA)	1.73	0.40–7.49	.46	Random	92.56	.00	0.50	0.60
Homozygous model (CC vs AA)	1.71	0.93–3.15	.08	Fixed	26.95	.25	0.83	0.60

### 3.5. Meta-analysis of IL23R rs10889677 polymorphism on AA risk

**Figure 5.**
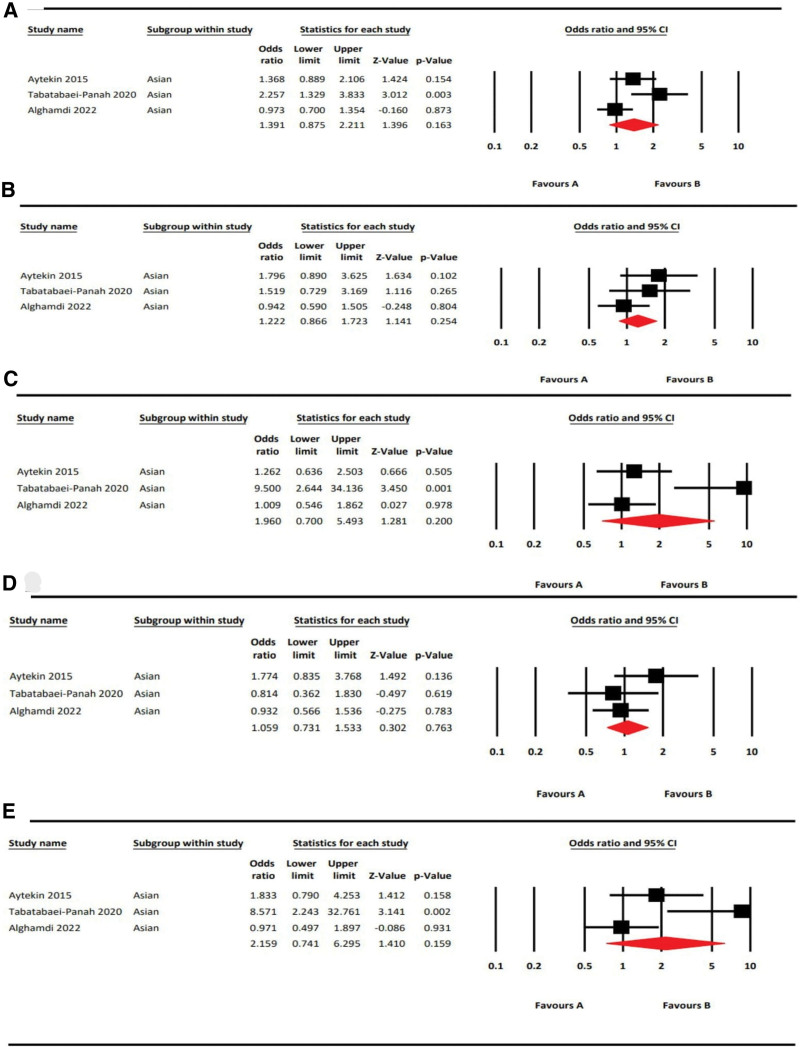
Forest plots of rs10889677 (IL23R) with AA risk in 5 genetic models. A: Allelic model (A vs C); B: Dominant model (AA + AC vs CC); C: Recessive model (AA vs AC + CC); D: Heterozygous model (AC vs CC) and E: Homozygous model (AA vs CC).

Table 7 summarizes the results of our meta-analysis of the association between the IL23R rs10889677 SNP and AA. Three articles were included in the meta-analysis consisting of 312 cases and 281 controls.^[16–18]^ Our analysis showed that, IL123R rs10889677 polymorphism did not have a risk effect in the allelic model, A vs C [OR = 1.39, 95%CI = 0.88–2.21, *P* = .16, *I*^2^ = 71.99%]; dominant model, AA + AC vs CC [OR = 1.22, 95%CI = 0.87–1.72, *P* = .25, *I*^2^ = 25.28%]; recessive model AA vs AC + CC [OR = 1.96, 95%CI = 0.70–5.49, *P* = .20, *I*^2^ = 79.58%], heterozygous model, AC vs CC [OR = 1.06, 95%CI = 0.73–1.53, *P* = .76, *I*^2^ = 18.62%]; homozygous model, AA vs CC [OR = 2.16, 95%CI = 0.74–6.30, *P* = .16, *I*^2^ = 75.77%]. The forest plots are shown in Figure 5. According to the ethnicity mentioned in the articles, the populations from Turkey, Iran, and Jordan were grouped as Asians.

**Table 7:** Association of rs10889677 (IL23R) with alopecia areata.

Model name	Association test			Heterogeneity test			Publication bias (*P* value)	
	Odds ratio(OR)	95% CI	*P* value	Model	I^2^	*P* value	Egger test	Begg-Mazumdar test
rs10889677 (IL23R)								
Allelic model (A vs C)	1.39	0.88–2.21	.16	Random	71.99	.03	0.08	0.12
Dominant model (AA + AC vs CC)	1.22	0.87–1.72	.25	Fixed	25.28	.26	0.18	0.60
Recessive model (AA vs AC + CC)	1.96	0.70–5.49	.20	Random	79.58	.01	0.01	0.12
Heterozygous model (AC vs CC)	1.06	0.73–1.53	.76	Fixed	18.62	.29	0.77	0.60
Homozygous model (AA vs CC)	2.16	0.74–6.30	.16	Random	75.77	.02	0.03	0.12

### 3.6. Publication bias

Begg-Mazumdar correlation test revealed no publication bias in any of the genetic models for all 4 SNPs. However, Egger regression test revealed a substantial publication bias for rs10889677 of IL23R gene in the recessive and homozygous models (Table 7). For these 2 genetic models, funnel plots were used to adjust the odds ratio. The supplementary files (Figs. S1, S2, S3, and S4, Supplemental Digital Content, http://links.lww.com/MD/L713, http://links.lww.com/MD/L714, http://links.lww.com/MD/L715, http://links.lww.com/MD/L716) illustrates all the funnel plots.

## 4. Discussion

We found a significant risk effect for rs3118470 of the IL2RA gene in the dominant and homozygous models with alopecia areata. However, for the other 3 SNPs, we could not find any statistically significant association. In a previous study conducted in the Chinese population, researchers found that the frequency of the C allele was substantially higher in the case group compared to controls.^[19]^ Moreover, Moravvej et al, reported that the C allele was significantly associated with developing AA in the Iranian population.^[27]^ This is in agreement with our findings where C allele was found to exhibit a risk effect in dominant and homozygous models for developing AA. Moreover, Redler et al also found that C allele was significantly associated with AA in a population with Caucasian ancestry.^[21]^ However, Alghamdi et al’s study contradicts these findings, claiming that there is no association of IL2RA gene SNP with AA in the Jordanian population.^[16]^

IL2RA acts as a receptor for IL2 and plays a key role in regulating the survival and proliferation of Treg cells.^[29]^ IL2RA is an immunological susceptibility locus for regulatory T-cell activation and proliferation.^[30]^ It has been hypothesized that mutation of IL2RA may disrupt the binding of IL2 and thus cause an imbalance in the immune response of Treg cells which may result in AA.^[31]^

According to Petukhova et al, a number of variables may interact to induce and promote immunological dysregulation in the AA pathogenesis. Strong evidence was discovered for genes involved in the development and maintenance of pro-inflammatory T helper cells (TH17), which act as functional antagonists of immunosuppressive Treg cells. Both IL-2 and its high-affinity receptor IL-2RA play important roles in Treg cell survival and proliferation.^[29]^ Studies need to be conducted to find out if rs3118470 acts as an expression quantitative trait locus for IL2RA and affects gene and protein expression.

For rs2275913 of the IL17A gene, we found no statistically significant association with AA in all 5 genetic models. This finding is similar to the findings of 2 previous articles where they reported no significant association of this polymorphism with AA in the Iranian and Jordanian populations.^[17,20]^ However, Seleit et al reported that there was a significant association with AA in the Egyptian population.^[28]^ A positive association was observed by Tojo et al between the number of interferon-producing cells (IL17) and the severity and progression of alopecia.^[32]^ However, our findings showed that there was no significant association found between IL17A gene and the development of AA. Nevertheless, further study is needed to determine how the rs2275913 polymorphism impacts mRNA and protein expression and risk in people with varied ethnic backgrounds and larger sample sizes.

We found no statistically significant association of rs3212227 with AA. This finding is consistent with 2 previous reports where no significant association of this SNP with AA was reported in the Turkish and Jordanian populations.^[18,20]^ Contrary to this, Tabatabaei-Panah et al reported that rs3212227 CC genotype was strongly linked to late-onset AA in the Iranian population and higher expression of IL12B was observed in patients compared to controls.^[17]^ Numerous immune-related conditions, such as type 1 diabetes, inflammatory bowel disease, asthma, rheumatoid arthritis, allergic rhinitis, and alopecia areata, have been linked to the IL12B polymorphism,^[33]^ however, the exact mechanism is yet to be identified.

For rs10889677 of the IL23R gene, we found no statistically significant association in all 5 genetic models. According to Aytekin et al and Eitan et al, there was no statistically significant association was detected for this SNP with AA in Turkish and Jordanian populations.^[16,18]^ In contrast, a positive association was reported in the Iranian population and late-onset AA was substantially correlated with the AA genotype of the IL23R gene polymorphisms.^[17]^ Helper T cell 17 (Th17) cell-mediated inflammation and the immune response depend on the IL23 receptor (IL23R), which binds to its cytokine, IL23.^[34,35]^ Numerous chronic disorders such as psoriasis, spondyloarthritis, Crohn disease, and ulcerative colitis have been associated with polymorphisms in the IL23R gene, which codes for the IL23 cytokine receptor.^[36–38]^ However, the role of IL23R gene in AA is yet to be identified. Future studies with larger sample sizes of people with different ethnicities are thus warranted. Moreover, gene and protein expression studies need to be conducted to elucidate the exact role of interleukin gene polymorphisms with alopecia areata. The clinical relevance of our meta-analysis is that we tried to narrow down and find out the overall impact of interleukin genes and the most relevant polymorphisms responsible for alopecia areata. This is of great importance as it will guide future interleukin family gene expression and protein expression studies and subsequently find out the target protein for the development of new drugs and clinical trials in patients with alopecia areata.

Our study has limitations. The number of case–control studies available is limited in alopecia areata. As a result, we could only do the meta-analysis for 4 SNPs of interleukin family genes. The association of the SNPs with alopecia areata can be population-specific but we could not perform a meta-analysis based on ethnicity as the required number of studies with specific ethnic backgrounds is unavailable. Future meta-analyses with larger sample sizes and specific ethnicity are thus warranted.

## 5. Conclusion

In summary, we performed a meta-analysis to identify the association between the SNPs of the Interleukin genes IL2RA, IL17A, IL12B, and IL23R (rs3118470, rs2275913, rs3212227, rs10889677) with the susceptibility of AA. Our study showed that polymorphisms of rs3118470 of the IL2RA gene were significantly associated with alopecia areata.

## Author contributions

**Conceptualization:** Zasia Hossain Tishe, Meherun Nessa Popy, Md Shaki Mostaid, Sanjana Shawkat.

**Data curation:** Zasia Hossain Tishe, Sanjana Shawkat, Meherun Nessa Popy.

**Formal analysis:** Zasia Hossain Tishe, Sanjana Shawkat, Meherun Nessa Popy.

**Investigation:** Ashfaq Ahmed, Zasia Hossain Tishe, Sanjana Shawkat, Meherun Nessa Popy.

**Methodology:** Md Shaki Mostaid.

**Resources:** Ashfaq Ahmed, Sadia Biswas Mumu.

**Software:** Ashfaq Ahmed, Sadia Biswas Mumu.

**Supervision:** Md Shaki Mostaid.

**Validation:** Mohd Nazmul Hasan Apu.

**Writing – original draft:** Zasia Hossain Tishe, Sanjana Shawkat, Meherun Nessa Popy.

**Writing – review & editing:** Mohd Nazmul Hasan Apu, Md Shaki Mostaid.

## Supplementary Material








