# Supplementary material for: Interleukin gene polymorphisms and alopecia areata: A systematic review and meta-analysis
Source: Medicine (Baltimore). 2024 Feb 23;103(8):e37300. doi: 10.1097/MD.0000000000037300 (PMC10883625; doi:10.1097/MD.0000000000037300)
Supplement: Supplementary file 3 [file medi-103-e37300-s003.docx]

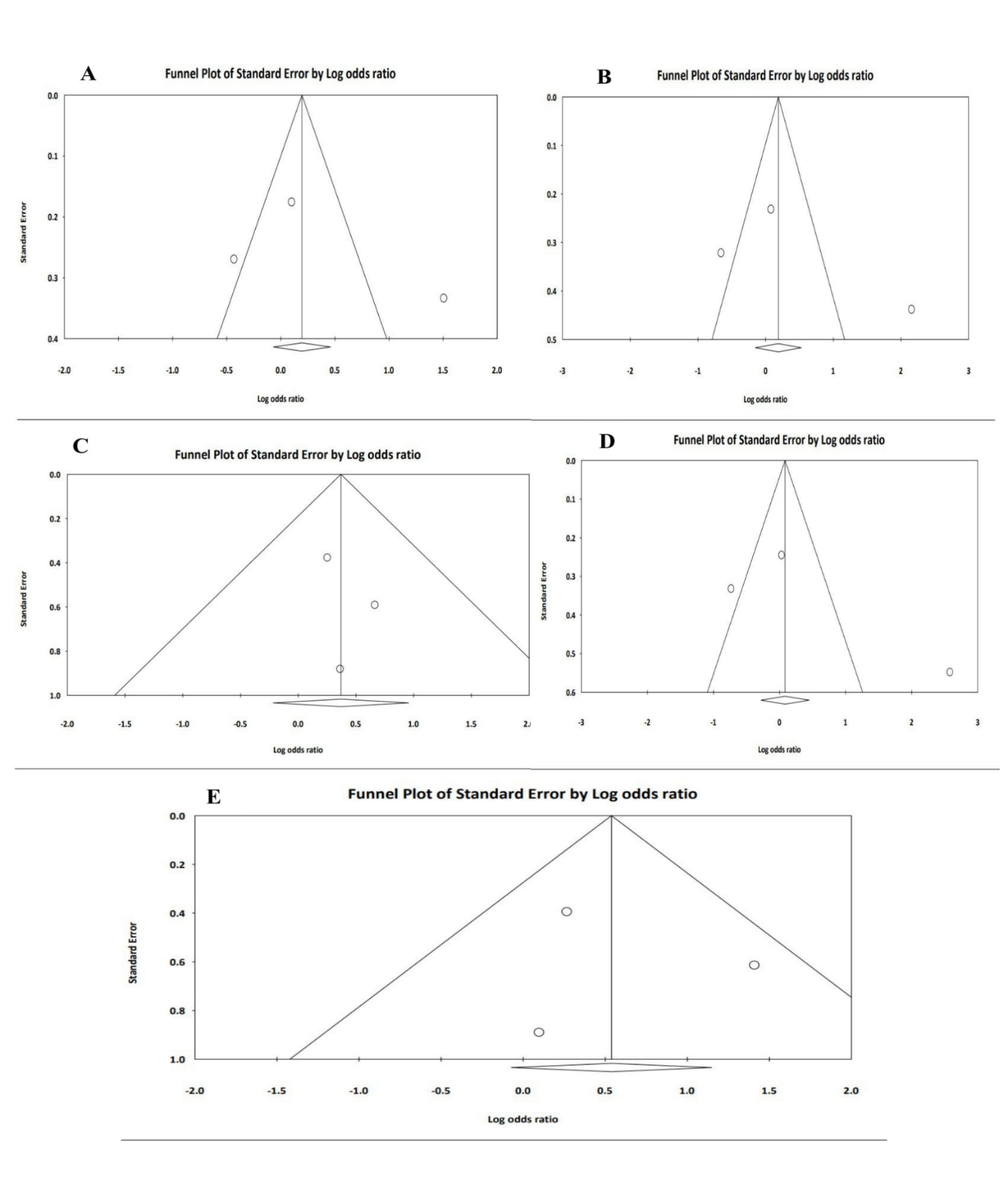


**Supplementary Figure S3:** Generated Funnel Plots in the Association of **rs3212227 (IL12B)** with Alopecia Areata. A= allelic model, B= dominant model, C= recessive model, D= heterozygous model, E= homozygous model.
