# Supplementary material for: Interleukin gene polymorphisms and alopecia areata: A systematic review and meta-analysis
Source: Medicine (Baltimore). 2024 Feb 23;103(8):e37300. doi: 10.1097/MD.0000000000037300 (PMC10883625; doi:10.1097/MD.0000000000037300)
Supplement: Supplementary file 4 [file medi-103-e37300-s004.docx]

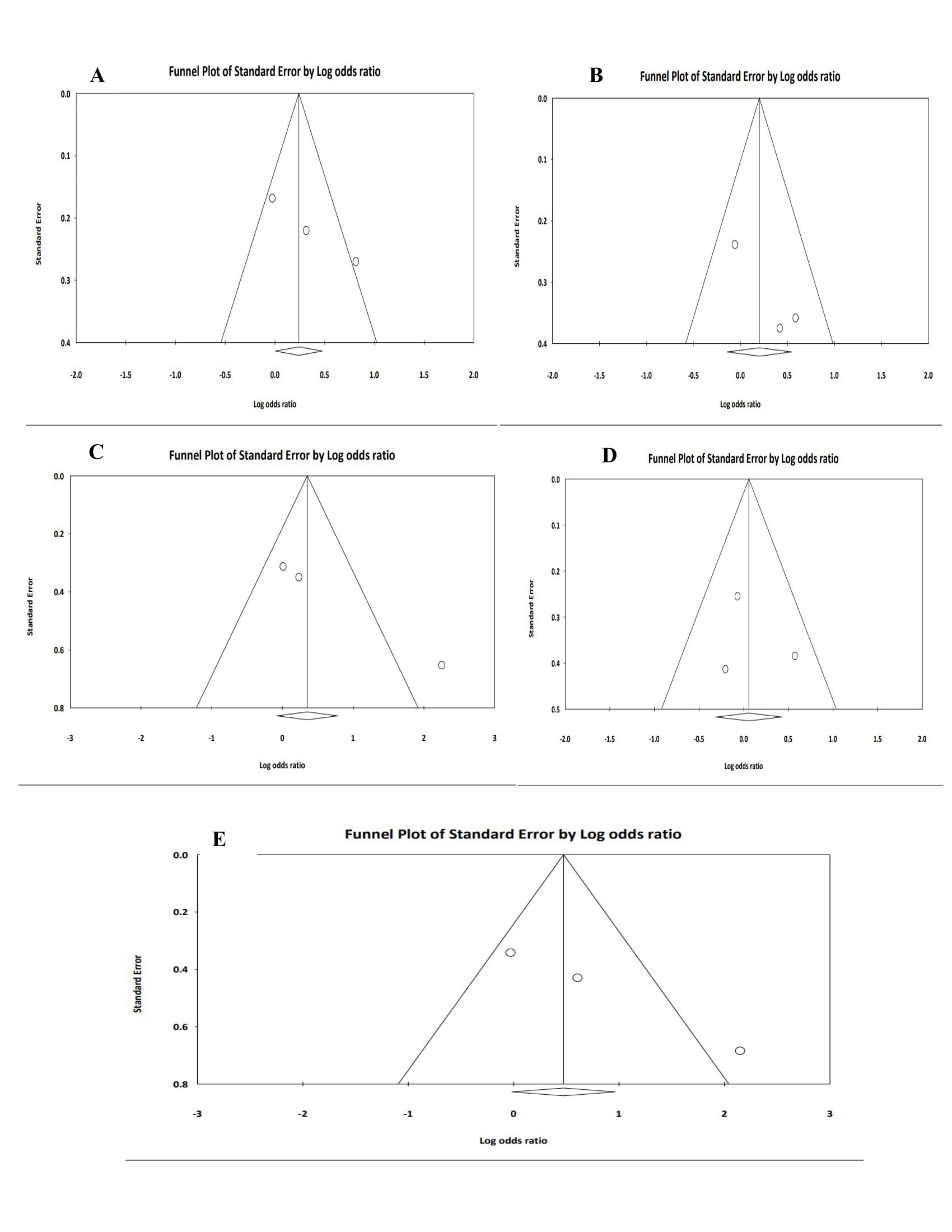


**Supplementary Figure S4:** Generated Funnel Plots in the Association of **rs10889677 (IL23R)** with Alopecia Areata. A= allelic model, B= dominant model, C= recessive model, D= heterozygous model, E= homozygous model.
